# Promoting higher-valent pediatric combination vaccines in China: challenges and recommendations for action

**DOI:** 10.1186/s40249-024-01181-9

**Published:** 2024-02-01

**Authors:** Jiuling Li, Shu Chen, Edwin Asturias, Shenglan Tang, Fuqiang Cui

**Affiliations:** 1 Global Health Research Center, Duke Kunshan University, Kunshan, Jiangsu China; 2 ARC Centre of Excellence in Population Ageing Research (CEPAR), University of New South Wales, Sydney, Australia; 3 School of Risk and Actuarial Studies, University of New South Wales, Sydney, Australia; 4 Department of Pediatrics, University of Colorado School of Medicine, Aurora, CO USA; 5 Center for Global Health, Colorado School of Public Health, Aurora, CO USA; 6 Department of Epidemiology, Colorado School of Public Health, Aurora, CO USA; 7 Duke Global Health Institute, Duke University, Durham, NC USA; 8 SingHealth Duke-NUS Global Health Institute, Duke-NUS, Singapore, Singapore; 9 Department of Laboratorial Science and Technology, School of Public Health, Peking University, Beijing, China; 10 Vaccine Research Center, School of Public Health, Peking University, Beijing, China

**Keywords:** Combination vaccine, National immunization program, Childhood immunization, Vaccine-preventable disease

## Abstract

Many countries have adopted higher-valent pediatric combination vaccines to simplify vaccination schedules and minimize health expenditures and social costs. However, China is conservative in the use of pediatric combination vaccines. By reviewing and synthesizing quantitative and qualitative data, in this commentary we identify gaps and challenges to combination vaccine use and make recommendations for promoting use of higher-valent pediatric combination vaccines in China. Challenges are in four dimensions: (1) legislation and regulation, (2) immunization schedule design, (3) vaccine awareness and price, and (4) research and development capacity. To optimize the use of combination vaccines to reduce vaccine-preventable disease burden, we make recommendations that address key challenges: (1) develop policies and regulations to strengthen enforcement of the Vaccine Administration Law and remove regulatory hurdles that hinder combination vaccine research and development, (2) establish an evidence-informed policy-making mechanism for combination vaccines, (3) resolve immunization schedule conflicts between monovalent and combination vaccines, and (4) implement effective interventions to increase vaccine awareness and reduce price.

**Graphical Abstract:**

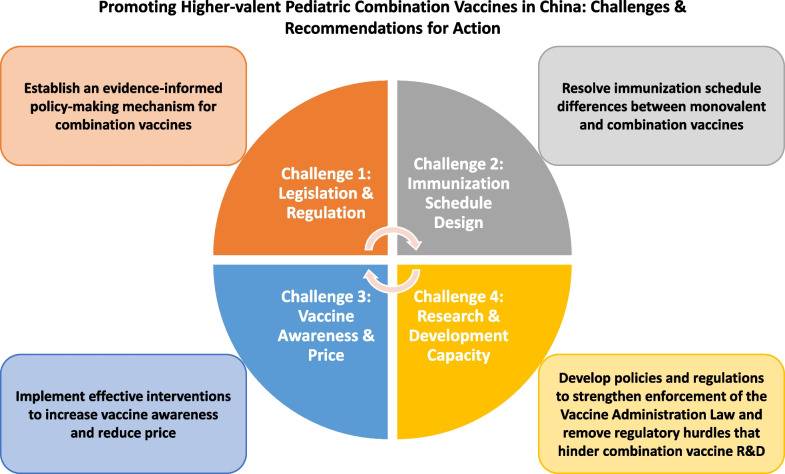

## Background

Diphtheria, tetanus, and pertussis (DTP) trivalent vaccines were invented in the 1940s [1] and continued to serve as a foundation of immunization programs. Fifty years later, antigens from poliovirus, hepatitis B (HepB) virus, and *Haemophilus influenzae* type b (Hib) were added to existing trivalent vaccines to make higher-valent vaccines. These higher-valent pediatric combination vaccines have demonstrated many advantages over monovalent, bivalent, and trivalent vaccines. Tetravalent, pentavalent, and hexavalent vaccines improve compliance and timeliness of vaccination [2], reduce healthcare professionals’ workloads [3] and risk of needlestick injuries [4], decrease the number of injections to save space for more new antigens in vaccination schedule, simplify immunization procedures [5], increase vaccination coverage [6], and minimize healthcare expenditures [7].

Due to these advantages, both developing and developed countries are promoting the use of higher-valent pediatric combination vaccines for improved cost-effectiveness and better health outcomes. As of 2018, pentavalent vaccines have been included in 132 countries’ National Immunization Programs (NIPs) or Expanded Programs on Immunization (EPIs) [8]. In 2019, hexavalent vaccines became available in more than 100 countries, with 35 countries had included hexavalent vaccines in their NIPs [9]. GAVI, the Vaccine Alliance, is an international organization that has contributed greatly to reducing the global burden of vaccine-preventable diseases (VPDs) by providing pentavalent vaccine to the 73 least-developed countries in the world. Between 2000 and 2022, full coverage with pentavalent vaccines rose from less than 1% to 82% in the 57 GAVI-supported countries [10]. China had achieved an overall coverage rate of 90% for NIP vaccines [11], yet it falls behind in the use, license granting mechanisms, and research and development of higher-valent pediatric combination vaccines. China’s NIP includes only two trivalent vaccines—diphtheria, tetanus, and acellular pertussis (DTaP) and measles, mumps, and rubella (MMR) vaccines, both of which were introduced in 2008 [12]. Moreover, since higher-valent pediatric combination vaccines are self-paid, non-NIP vaccines in China, their coverage levels remain low overall, and lowest in areas with poorer socioeconomic development [13–15].

In this commentary, we (1) identify gaps in inclusion and coverage of relevant pediatric combination vaccines in NIPs, contrasting China with selected developing and developed countries, (2) analyze the main challenges of promoting combination vaccines, and (3) propose actions to improve use of combination vaccines in China that are aligned with strategic priority goals of Immunization Agenda 2030 (IA2030) relating to access to vaccines, equitable and high vaccine coverage, and innovation in vaccine use and development.

We collected, reviewed, and synthesized both quantitative and qualitative data from the English and Chinese scientific literature, policy documents issued by governments, position papers and research reports generated by international organizations, original databases such as the WHO Immunization Data Portal, and the grey literature. We focused on vaccines containing antigens that shows great efficacy and safety and have been used extensively for decades, including DTaP, Hib, IPV, HepB, and MMR. We obtained relevant vaccine prices and information about immunization schedules from the U.S. Center for Disease Control and Prevention (US CDC), the Pan American Health Organization (PAHO), UNICEF, and Chinese government websites. We selected several high-income and low- and middle-income countries (LMICs) that were early adopters of pediatric combination vaccines and have achieved relatively high coverage for an in-depth, comparative analysis.

### Gaps in the use of higher-valent pediatric combination vaccines between China and other countries

Pediatric combination vaccines have been included in many developed countries’ NIPs [4], which means that they are funded by government and provided to the public free of charge. These vaccines are often updated with higher-valent vaccines as they become available. The U.K. provides routine immunization services through the National Health Service (NHS). In 2017, NHS replaced DTaP-IPV-Hib pentavalent vaccine with DTaP-HepB-IPV-Hib hexavalent vaccine, providing infants born after August 1, 2017 with protection from Hepatitis B virus infection [16] (Table 1). The U.S. federal government-funded Vaccines for Children (VFC) program provides several pediatric combination vaccines free of charge to families of children who are Medicaid-enrolled, uninsured, American Indian/Alaska Native, or underinsured and served in a federally-qualified health center [17]. GAVI provides DTwP-Hib-HepB pentavalent vaccine for children in low-income countries to boost low uptake of Hib and HepB vaccines by making them part of routine immunization programs [18]. Malaysia, a pioneer in using higher-valent pediatric combination vaccines among middle-income countries ineligible for GAVI support, introduced DTwP-Hib-HepB vaccine in its National Immunization Program in 2006, replacing it with DTaP-Hib-HepB vaccine in 2008, and updating to hexavalent DTaP-HepB-IPV-Hib vaccine in 2020 [19]. The Ministry of Health (MoH) of Malaysia acts as funder, provider, and regulator for all routine immunizations [20]. Brazil replaced DTP vaccines with DTP-Hib in 2003 and upgraded this tetravalent vaccine to a pentavalent vaccine in 2012 [21]—all purchased through the PAHO Strategic Fund by Brazil’s MoH [22].

**Table 1:** Higher-valent combination vaccines (MMR + & DTP +) covered by the National Immunization Programs in China and selected countries

Country	Tetravalent vaccines	Pentavalent vaccines	Hexavalent vaccines
China	–	–	–
U.S.	DTaP-IPV MMR	DTaP-IPV-Hib DTaP-IPV-HepB	DTaP-HepB-IPV-Hib
U.K.	DTaP-IPV	–	DTaP-HepB-IPV-Hib
Germany	MMR	–	DTaP-HepB-IPV-Hib
Singapore	–	DTaP-IPV-Hib	–
Malaysia	–	–	DTaP-HepB-IPV-Hib
Brazil	–	DTwP-Hib-HepB	–
Cambodia (GAVI-supported)	–	DTwP-Hib-HepB	–

– means not applicable

*DTaP-IPV* Diphtheria, tetanus, acellular pertussis, and polio; *MMR* Measles, mumps, and rubella; *DTaP-Hib* Diphtheria, tetanus, acellular pertussis, and *Haemophilus influenzae* type b; *DTaP-IPV-HepB* Diphtheria, tetanus, acellular pertussis, polio and hepatitis B; *DTaP-HepB-IPV-Hib* Diphtheria, tetanus, acellular pertussis, hepatitis B, polio, and *Haemophilus influenzae* type b; *DTwP-Hib-HepB* Diphtheria, tetanus, whole cell pertussis, *Haemophilus influenzae* type b, and hepatitis B

**Fig. 1:**
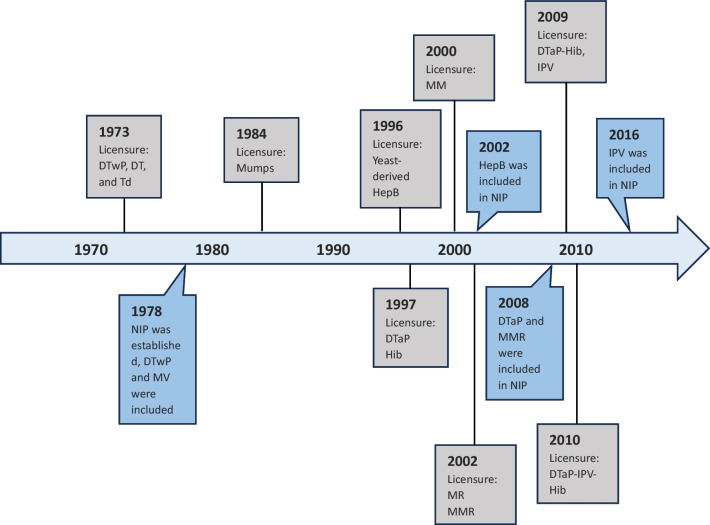
Timeline of relevant vaccine licensures and inclusions into China’s National Immunization Programs system. *DTwP* Diphtheria, tetanus, and whole cell pertussis; *DT* Diphtheria, tetanus; *Td* Tetanus-diphtheria; *HepB* Hepatitis B; *DTaP* Diphtheria, tetanus, acellular pertussis; *Hib Haemophilus influenzae* type b; *MM* Measles and mumps; *MMR* Measles, mumps, and rubella; *MR* Measles and rubella; *NIP* National Immunization Program; *DTaP-Hib* Diphtheria, tetanus, acellular pertussis, and *Haemophilus influenzae* type b; *IPV* Inactivated poliovirus vaccine; *DTaP* Diphtheria, tetanus, and acellular pertussis; *DTaP-IPV-Hib* Diphtheria, tetanus, acellular pertussis, polio, and *Haemophilus influenzae* type b

China remains conservative in the inclusion of higher-valent pediatric combination vaccines into NIPs in comparison to other countries (Table 2). China’s NIP was launched in 1978 in response to a call from the World Health Organization (WHO). Diphtheria, tetanus, and whole cell pertussis (DTwP) vaccine and live attenuated measles vaccine were included in the original NIP schedule and were subsequently replaced by DTaP and MMR vaccines in 2008 (Fig. 1). Although dramatic declines in morbidity occurred in most of the 11 childhood VPDs targeted in routine immunization [23], other than IPV in 2016, China has not included any new vaccines in the NIP system over the past 15 years (Table 3), vaccines such as pneumococcal conjugate vaccines (PCV), human papillomavirus (HPV) and rotavirus vaccines that have been widely used in many countries are not provided through China’s NIP, and is the only WHO Member State that has not included Hib vaccine in the NIP [24]. Pediatric tetravalent vaccine and IPV were not licensed in China until 2009, and a pentavalent vaccine was only approved in 2010. At present, no hexavalent vaccine is available. All higher-valent pediatric combination vaccines are categorized as non-NIP vaccines, meaning that they are family-paid optional vaccines that can substitute for their lower-valent program vaccine equivalents while including more antigens.

**Table 2:** Higher-valent pediatric combination vaccines and the timeline included in the NIPs in selected countries

Category	U.S.	U.K.	Germany	Singapore	Malaysia	Brazil	Cambodia	China
Tetravalent vaccines	MMR (2006)	DTaP-IPV (2004)	MMR (2004)	–	–	MMR (2013)	–	–
	DTaP-IPV (2008)		DTaP-IPV (2006)					
Pentavalent vaccines	DTaP-HepB-IPV (2003)	–	–	DTaP-IPV-Hib (2016)	–	DTwP-Hib-HepB (2012)	DTwP-Hib-HepB (2005)	–
	DTaP-IPV-Hib (2008)							
Hexavalent vaccines	DTaP-IPV-Hib-HepB (2019)	DTaP-IPV-Hib-HepB (2017)	DTaP-IPV-Hib-HepB (2000)	–	DTaP-IPV-Hib-HepB (2020)	–	–	–

– means not applicable

*MMR* Measles, mumps, and rubella; *DTaP-IPV* Diphtheria, tetanus, acellular pertussis, and polio; *DTaP-HepB-IPV* Diphtheria, tetanus, acellular pertussis, hepatitis B, and polio; *DTaP-IPV-Hib* Diphtheria, tetanus, acellular pertussis, polio, and *Haemophilus influenzae* type b; *DTwP-Hib-HepB* Diphtheria, tetanus, whole cell pertussis, *Haemophilus influenzae* type b, and hepatitis B; *DTaP-IPV-Hib* Diphtheria, tetanus, acellular pertussis, polio, and *Haemophilus influenzae* type b; *DTaP-IPV-Hib-HepB* Diphtheria, tetanus, acellular pertussis, polio, *Haemophilus influenzae* type b, and hepatitis B

**Table 3:** China’s current NIP schedule and coverage (as of 2021)

Target antigen	Vaccine	Age															Coverage of antigen (%)
		At birth	1 m	2 m	3 m	4 m	5 m	6 m	8 m	9 m	18 m	2 y	3 y	4 y	5 y	6 y	
HepB virus	HepB	1	2					3									99.2
*Mycobacterium tuberculosis*	BCG	1															99.7
Poliomyelitis virus	IPV			1	2												99.2
	bOPV					3								4			
Diphtheria, tetanus, pertussis	DTaP				1	2	3				4						98.8
	DT															5	
Measles, mumps, rubella	MMR								1		2						99.1
Japanese encephalitis virus	JE-L								1			2					99.1
	JE-I								1 and 2			3				4	
*Neisseria meningitidis*	MPSV-A																99.6
	MPSV-AC												3			4	99.2
HepA virus	HepA-L										1						99.2
	HepA-I										1	2					–

m means months, y means years, – means not applicable

*HepB* Hepatitis B; *BCG* Bacillus Calmette-Guérin; *IPV* Inactivated poliovirus vaccine; *bOPV* Bivalent oral polio vaccine; *DTaP* Diphtheria, tetanus, and acellular pertussis; DT Diphtheria, tetanus; *MMR* Measles, mumps, and rubella; *JE-L* Live-attenuated Japanese encephalitis vaccine; *JE-I* Inactivated Japanese encephalitis vaccine; *MPSV-A* Group A meningococcal polysaccharide vaccine; *MPSV-AC* Group A and Group C meningococcal polysaccharide vaccine; *HepA-L* Live-attenuated hepatitis A; *HepA-I* Inactivated hepatitis A

Coverage of non-NIP vaccines is generally low in China, and recent research findings show that pediatric tetravalent and pentavalent have the lowest coverage levels of all non-NIP vaccines. Among 343 children whose families could have opted for a tetravalent vaccine, only 0.58% elected to pay for the tetravalent vaccine, and among 171 children whose family could have opted for a pentavalent vaccine, only 3.51% did so [13]. Coverage of higher-valent pediatric combination vaccines demonstrates regional disparities, as areas with higher socioeconomic development achieve higher vaccination coverage. In a study conducted in Beijing among 480 children 0 to 3 years of age, coverage of the first dose of pentavalent vaccine was 12.08% [25], a finding that is consistent with results (11.81%) from another Beijing study [26]. In regions with lower socioeconomic development, coverage of pentavalent vaccine ranges from 3.64% to 9.57% [14, 15].

In contrast, countries that have included higher-valent pediatric combination vaccines in their NIPs have significantly higher coverage of combination vaccines. For example, NHS data show that hexavalent vaccine coverage was 93.5% among 24-month-old children in 2022 [27]. In Malaysia, the National Health and Morbidity Survey (NHMS) showed that full-series coverage with pentavalent vaccines was 86.4% in 2016 [28]. Low-income countries also achieve relatively high levels of higher-valent vaccine coverage. Cambodia’s Demographic and Health Survey shows that 84.1% of children 12–23 months of age received three doses of DPT-HepB-Hib vaccine in 2022 [29].

### Challenges promoting higher-valent pediatric combination vaccines in China

Challenges promoting higher-valent pediatric combination vaccines in China arise from several aspects, including regulation and legislation, immunization program design, vaccine awareness and acceptance, research and development (R&D), and vaccine supply.

First, although the Vaccine Administration Law of 2019 encourages combination vaccine innovations, there has been deficient policy support to accelerate implementation. Before July 2022, pharmaceutical companies in China were required to have monovalent vaccines individually approved before producing combination vaccines that contain the same antigens as components [30]. After establishment of the *Rules on the Administration of Vaccine Manufacturing and Distribution* in July 2022, contract manufacturing of combination vaccines by different pharmaceutical companies was allowed but can only be approved when both the grantor and the grantee have been evaluated and verified by the National Medical Products Administration [31]. No new combination vaccines have entered the market subsequent to the new rules. Local CDC experts expressed concern about being able to attribute adverse events following immunization to specific vaccines and favor monovalent vaccines because they can more easily determine which antigens causes which adverse event and help attribute responsibility for adverse event between CDCs and healthcare facilities [32].

Second, including higher-valent pediatric combination vaccines in China’s National Immunization Program is time-consuming and requires solutions to potential immunization schedule conflicts. In 2019, the Vaccine Administration Law mandated the necessity of evidence-based recommendations to the National Health Commission (NHC) for inclusion of new vaccines into China’s National Immunization Program [33]. For example, although the National Immunization Advisory Committee (NIAC) has rendered opinions on Hib vaccines in 2018 and 2019 [34], evidence required for program inclusion is still being collected, which includes assessment of disease burden, cost-effectiveness, safety and effectiveness of the vaccine, and assured domestic supply. Particularly, the evidence collection is hindered by the lack of high-quality data on the disease burden caused by Hib infections, which is underestimated in China due to the wide and over-utilization of antibiotics. The process of evidence gathering as well as decision making has therefore been slow.

In addition, there are differences in the monovalent HepB schedule and HepB-containing combination vaccine schedule (Table 4). Research and careful program evaluation have proven that providing timely HepB monovalent vaccine birth doses to newborns is critically important for preventing mother-to-child transmission of hepatitis B virus. Since the inclusion of monovalent HepB vaccines in China’s National Immunization Program, the prevalence of chronic hepatitis B virus infection among children under 5 years of age has decreased by over 95%, from 9.7% to less than 1%, and the seroprevalences of hepatitis B surface antigen among children aged 5–9 and 10–14 years have decreased by 86% and 72%, respectively [35]. In accordance with China’s current immunization schedule, newborns are given three doses of monovalent HepB vaccine: at birth, 1 month, and 6 months of age. HepB-containing combination vaccines, however, are given in different schedules. Hexavalent HepB-containing combination vaccines are given at 6 weeks, 10 weeks, and 14 weeks of age, or at 2 months, 4 months, and 6 months of age [36]. Resolving the differences in the schedule while maintaining effective infection prevention levels are challenging for China, a country with a high burden of hepatitis B [37].

**Table 4:** Immunization schedules of U.S. FDA-licensed higher-valent combination vaccines

Vaccine	Trade name (year licensed)	Age range	Routinely recommended ages
DTaP-IPV	Kinrix (2008)	4–6 years	5th dose of DTaP, and 4th dose of IPV between 4 and 6 years of age
DTaP-IPV	Quadracel (2015)	4–6 years	5th dose of DTaP, and 4th or 5th dose of IPV between 4 and 6 years of age
DTaP-IPV-Hib	Pentacel (2008)	6 weeks–4 years	4-dose series at 2, 4, 6, and 15–18 months of age
DTaP-HepB-IPV	Pediarix (2002)	6 weeks–6 years	3-dose series at 2, 4, and 6 months of age
DTaP-IPV-Hib-HepB	Vaxelis (2018)	6 weeks–4 years	3-dose series at 2, 4, and 6 months of age

*DTaP-IPV* Diphtheria, tetanus, acellular pertussis, and polio; *DTaP-IPV-Hib* Diphtheria, tetanus, acellular pertussis, polio, and *Haemophilus influenzae* type b; *DTaP-HepB-IPV* Diphtheria, tetanus, acellular pertussis, hepatitis B, and polio; *DTaP-IPV-Hib-HepB* Diphtheria, tetanus, acellular pertussis, polio, *Haemophilus influenzae* type b, and hepatitis B

Fourth, low awareness and high price are other important hurdles behind the low uptake of the higher-valent combination vaccines [38]. A recent study showed general low awareness of non-NIP vaccines, and awareness of pediatric pentavalent vaccines was lowest [39]. Based on a study conducted in the well-developed Dongcheng District of Beijing, among 183 parents, only 21.31% (39) had heard of pentavalent vaccine and only 10.93% knew that higher-valent pediatric combination vaccines could substitute for program vaccines [40]. In underdeveloped regions, awareness of higher-valent pediatric combination vaccines is lower due to the lack of education and immunization campaigns and insufficient knowledge of healthcare providers [41]. A systematic review showed that concerns about vaccine safety, reactogenicity, efficacy, effectiveness, and protection influence vaccine hesitancy toward non-NIP vaccines in China, but high price plays a more important role in discouraging Chinese parents to vaccinate their children with family-paid combination vaccines [41]. The latest Shanghai municipal government contract price of a dose of domestic tetravalent vaccine is CNY 368 (USD 51), and the price of a dose of imported pentavalent vaccine is CNY 599 (USD 83) [42]. Both of these prices exceed the global average price and pose heavy financial burdens for Chinese parents [43–45] (Table 5).

**Table 5:** China, PAHO, and U.S. CDC contract prices per dose in 2022

Vaccine type	China	PAHO	GAVI	U.S. CDC
Tetravalent vaccines	DTaP-Hib USD 51.00	DTaP-IPV USD 13.00	DTaP-HepB USD 0.69 (2012) DTaP-Hib USD 0.69 (2009)	DTaP-IPV USD 46.00–47.00 MMRV USD 165.00
Pentavalent vaccines	DTaP-IPV-Hib USD 83.00	DTaP-IPV-Hib USD 16.00	DTaP-HepB-Hib USD 0.75–1.15	DTaP-IPV-Hib USD 68.00 DTaP-IPV-HepB USD 64.00
Hexavalent vaccines	N/A	DTaP-HepB-IPV-Hib USD 21.00	DTaP-HepB-IPV-Hib USD 2.85–4.90 (2024)	DTaP-HepB-IPV-Hib USD 98.00

*DTaP-Hib* Diphtheria, tetanus, acellular pertussis, and *Haemophilus influenzae* type b;*DTaP-IPV* Diphtheria, tetanus, acellular pertussis, and polio; *DTaP-HepB* Diphtheria, tetanus, acellular pertussis, hepatitis B; *DTaP-IPV-Hib* Diphtheria, tetanus, acellular pertussis, polio, and *Haemophilus influenzae* type b; *DTwP-Hib-HepB* Diphtheria, tetanus, whole cell pertussis, *Haemophilus influenzae* type b, and hepatitis B; *DTaP HepB-IPV-Hib-* Diphtheria, tetanus, acellular pertussis, hepatitis B, polio, and *Haemophilus influenzae* type b

Lastly, Chinese pharmaceutical companies face research and development bottlenecks when developing higher–valent pediatric combination vaccines. Most combination vaccines, including DTaP-containing vaccines that are covered by the National Immunization Program, undergo a copurification process in manufacturing, during which thimerosal is added as a preservative [46]. However, thimerosal has a detrimental effect on IPV antigens, which can cause the poliovirus capsid to lose antigenicity [47]. The only domestic tetravalent vaccine is produced by Beijing Minhai Biotechnology Co., Ltd., which uses the same process as the U.S. and Europe, whose antigen components are separated before purification [48]. China only has imported pentavalent vaccines, and the volume of vaccine allocated to the Chinese market by vaccine companies, the process of customs clearance, and the timing of import inspection can affect timeliness of importation and supply. Each batch of imported vaccines is required to pass an inspection period of 3–6 months, which occupies approximately 25% of the vaccine shelf life (usually 2 years). Imported vaccines often fail to obtain certificates for release [49]. Thus, both the supply of domestic tetravalent and of imported pentavalent vaccines cannot meet the demand despite the low awareness of these vaccines among the general public.

## Recommendations

We propose several recommendations to overcome the above challenges and promote higher-valent pediatric combination vaccines in China.

First, develop and implement supporting policies that strengthen enforcement of the Vaccines Administration Law’s articles on combination vaccines. For example, Article 14 states that the State shall make research and development plans according to such factors as prevalence of diseases and population immunity and arrange necessary funds to support the development of novel vaccines such as combined polyvalent vaccines. Policy or regulatory actions could guide developers and manufacturers to produce concrete research and development plans over a given timeframe. Additionally, these actions could work to encourage combination vaccine development with accelerated approval pathways for licensure, develop proper intellectual property protections that incentivize innovation while not harming the vaccine accessibility, or support a regulatory needs assessment for actions that accelerate combination vaccine development and approval. Specifically, key regulatory hurdles should be removed to promote clinical trials of vaccine co-administration and simplify vaccine development and registration procedures. For instance, in China, applicants and marketing authorization holders (MAH) must be the same entity and are constrained to be pharmaceutical companies or research institutions that have obtained relevant product registration certifications. While in Europe and the U.S., applicants can be any individual, company, research institute, or organization. During the marketing authorization stage, the European Union (EU) allows applicants to submit previously awarded Vaccine Antigen Master File (VAMF) certificates that contain all relevant information of biological, pharmaceutical, and chemical nature for a given vaccine antigen if partial antigenic components of the new combination vaccines are identical to the vaccines from the same marketing authorization applicant or MAH [50]. The Center for Biologics Evaluation and Research (CBER) of U.S. Food and Drug Administration (FDA) permits a “case-by-case” approach to discuss use of technical information on the marketed component antigens with the applicants when approving new combination vaccines [51]. Actions taken by National Medical Products Administration (NMPA) thus far, such as allowing a combination vaccine maker to include antigens made by different manufacturers, clearly favor development of combination vaccines [31]. Through strategic regulation and policy, NMPA and NHC could use their power to accelerate combination vaccine development.

Second, a National Immunization Advisory Committee (NIAC) technical working group should be established and functioned to support evidence-informed policy-making for pediatric combination vaccines. The term of reference and working mechanism of NIAC need to be clearly defined. The technical group could be composed of NIAC members, public health professionals, academic experts, regulators, and clinicians. To ensure effectiveness of such a technical work group, voices from senior leaders of key stakeholders such as NHC, NMPA, and the Ministry of Finance should be included. NIAC could play an important role of suggesting combination vaccines that would be good for the National Immunization Program and for children by collecting and synthesizing high-quality evidence on disease burden, vaccine efficacy, safety, and cost-effectiveness. NIAC could review the entire NIP schedule to identify potential combination vaccines most favorable for program efficiency and effectiveness. NIAC could hold sessions on combination vaccines that include presentations by manufacturers and key stakeholders for a comprehensive assessment of the necessity of promoting combination vaccines and make a feasible plan to prioritize different combination vaccines step by step. For example, IPV-included combination vaccines, such as DTaP-IPV-Hib or DTaP-HepB-IPV-Hib could reduce injections, reduce vaccination clinic visits, and keep polio population immunity high. Both combination vaccines are in use globally and are producing good results, for example, nearly 100% trial enrollees achieved seroprotection against target antigens [52, 53].

Third, national immunization schedule needs to be regularly updated to resolve immunization schedule differences between monovalent and combination vaccines based on disease burden, clinical effectiveness, and international experience and research. The immunization schedule difference between monovalent HepB and HepB-containing combination vaccines is a good example. Considering the high disease burden of hepatitis B in China, it is essential to continue using the monovalent HepB birth dose and conducting evaluations to ensure that a schedule with the birth dose followed by a hexavalent HepB-containing combination vaccine does not lead to breakthrough maternal to child transmission of hepatitis B virus. Good lessons could be learned from other countries such as the U.K., where babies born to hepatitis B negative women are given a single dose of a monovalent hepatitis B vaccine before babies are discharged from the hospital while babies born to mothers who tested positive for hepatitis B virus surface antigen receive a total of six doses of HepB-containing vaccines between birth and 12 months of age: at birth (HepB monovalent), 4 weeks (HepB monovalent), 8 weeks (hexavalent), 12 weeks (hexavalent), 16 weeks (hexavalent), and 12 months (HepB monovalent) [54].

Lastly, improve public vaccine awareness and reduce vaccine price via comprehensive strategies. Organized, regular trainings for health professionals can advance their knowledge of higher-valent pediatric combination vaccines and to provide incentives to improve service quality. As a result, professionals will be able to communicate key advantages of these vaccines to parents and increase uptake in their children. Tailored health education interventions should be developed to address divergent concerns among parents. Possible solutions to reduce the price and out-of-pocket payments include joint procurement of the vaccines at a reasonable price and utilizing multiple financing channels, especially the medical insurance fund, to cover the cost. Developed regions can take the lead in launching pilot programs that enable residents to use the balance in their basic medical insurance account to pay for non-NIP vaccines for family members or provide government-subsidized health plan benefits to cover vaccine expenses.

## Conclusions

China has one of the highest burdens of childhood infectious diseases in the world. Although coverage with the current program vaccines is high, China could include more WHO-recommended vaccines in the National Immunization Program by embracing the use of higher-valent pediatric combination vaccines. Huge gaps exist in the development and use of higher-valent pediatric combination vaccines between China and other countries, irrespective of socioeconomic level. There is an urgent need to optimize China’s National Immunization Program, enhance vaccine awareness and acceptance, and encourage innovation, as we have proposed above, to promote the use of higher–valent combination vaccines and help reduce VPD morbidity and mortality.

## Data Availability

Data sharing is not applicable to this article as no datasets were generated or analyzed during the current study.

## Abbreviations

BCG: Bacillus Calmette-Guérin
bOPV: Bivalent oral polio vaccine
CBER: Center for Biologics Evaluation and Research
CDC: Center for Disease Control and Prevention
DT: Diphtheria, tetanus
DTaP: Diphtheria, tetanus, and acellular pertussis
DTaP-HepB-IPV: Diphtheria, tetanus, acellular pertussis, hepatitis B, and polio
DTaP-Hib: Diphtheria, tetanus, acellular pertussis, and *Haemophilus influenzae* type b
DTaP-IPV: Diphtheria, tetanus, acellular pertussis, and polio
DTaP-IPV-Hib: Diphtheria, tetanus, acellular pertussis, polio, and *Haemophilus influenzae* type b
DTaP-IPV-Hib-HepB: Diphtheria, tetanus, acellular pertussis, polio, *Haemophilus influenzae* type b, and hepatitis B
DTP: Diphtheria, tetanus, and pertussis
DTwP: Diphtheria, tetanus, and whole cell pertussis
DTwP-Hib-HepB: Diphtheria, tetanus, whole cell pertussis, *Haemophilus influenzae* type b, and hepatitis B
EPI: Expanded Program on Immunization
EU: European Union
FDA: Food and Drug Administration
GAVI: Vaccine Alliance
Hib: *Haemophilus Influenzae* type b
HepA-L: Live-attenuated hepatitis A
HepA-I: Inactivated hepatitis A
HepB: Hepatitis B
HPV: Human papillomavirus
IPV: Inactivated poliovirus vaccine
JE-L: Live-attenuated Japanese encephalitis vaccine
JE-I: Inactivated Japanese encephalitis vaccine
MAH: Marketing Authorization Holder
MM: Measles and mumps
MMR: Measles, mumps, and rubella
MoH: Ministry of Health
MPSV-A: Group A meningococcal polysaccharide vaccine
MPSV-AC: Group A and Group C meningococcal polysaccharide vaccine
MR: Measles and rubella
MV: Measles virus
NHMS: National Health and Morbidity Survey
NHC: National Health Commission
NHS: National Health Service
NIAC: National Immunization Advisory Committee
NIP: National Immunization Program
NMPA: National Medical Products Administration
R&D: Research and Development
Td: Tetanus-diphtheria
PAHO: Pan American Health Organization
PCV: Pneumococcal conjugate vaccine
VAMF: Vaccine Antigen Master File
VFC: Vaccines for children
VPD: Vaccine-preventable disease
WHO: World Health Organization
